# Lacosamide dosing in patients receiving continuous renal replacement therapy

**DOI:** 10.1186/s40560-023-00700-4

**Published:** 2023-11-09

**Authors:** Weerachai Chaijamorn, Sathian Phunpon, Thanompong Sathienluckana, Taniya Charoensareerat, Sutthiporn Pattharachayakul, Dhakrit Rungkitwattanakul, Nattachai Srisawat

**Affiliations:** 1 Department of Pharmacy Practice, Faculty of Pharmaceutical Sciences, Chulalongkorn University, Pathum Wan, Bangkok, 10330 Thailand; 2 Faculty of Pharmacy, Siam University, Bangkok, Thailand; 3 Department of Clinical Pharmacy, Faculty of Pharmaceutical Sciences, Prince of Songkla University, Songkhla, Thailand; 4 Department of Clinical and Administrative Pharmacy Sciences, College of Pharmacy, Howard University, Washington, DC USA; 5 Division of Nephrology, Department of Medicine, Faculty of Medicine, Chulalongkorn University and King Chulalongkorn Memorial Hospital, Bangkok, Thailand; 6 Excellence Center for Critical Care Nephrology, King Chulalongkorn Memorial Hospital, Bangkok, Thailand; 7 Critical Care Nephrology Research Unit, Faculty of Medicine, Chulalongkorn University, Bangkok, Thailand; 8 Academic of Science, Royal Society of Thailand, Bangkok, Thailand; 9 Tropical Medicine Cluster, Chulalongkorn University, Bangkok, Thailand; 10 Center for Critical Care Nephrology, The CRISMA Center, Department of Critical Care Medicine, University of Pittsburgh School of Medicine, Pittsburgh, PA USA

**Keywords:** Lacosamide, Pharmacokinetics, Drug dosing, Critically ill patients, Continuous renal replacement therapy

## Abstract

**Background:**

Lacosamide is one of the anticonvulsants used in critically ill patients. This study aimed to suggest appropriate lacosamide dosing regimens in critically ill patients receiving continuous renal replacement therapy (CRRT) via Monte Carlo simulations.

**Methods:**

Mathematical models were created using published demographic and pharmacokinetics in adult critically ill patients. CRRT modalities with different effluent rates were added into the models. Lacosamide regimens were evaluated on the probability of target attainment (PTA) using pharmacodynamic targets of trough concentrations and area under the curve within a range of 5–10 mg/L and 80.25–143 and 143–231 mg*h/L for the initial 72 h-therapy, respectively. Optimal regimens were defined from regimens that yielded the highest PTA. Each dosing regimen was tested in a group of different 10,000 virtual patients.

**Results:**

Our results revealed the optimal lacosamide dosing regimen of 300–450 mg/day is recommended for adult patients receiving both CRRT modalities with 20–25 effluent rates. The dose of 600 mg/day was suggested in higher effluent rate of 35 mL/kg/h. Moreover, a patient with body weight > 100 kg was less likely to attain the targets.

**Conclusions:**

Volume of distribution, total clearance, CRRT clearance and body weight were significantly contributed to lacosamide dosing. Clinical validation of the finding is strongly indicated.

## Introduction

Lacosamide is one of the newer antiepileptic agents. It has been widely used in clinical practice for focal (partial) onset seizure management in both monotherapy, and adjunctive therapy for primary generalized tonic–clonic seizures [1–3]. In addition, it can be utilized as an alternative antiepileptic agent for status epilepticus [2]. The effectiveness and safety profiles of lacosamide have been published in various randomized controlled studies [4, 5]. Lacosamide has an advantage for clinical use due to its linear pharmacokinetic property [6, 7]. The medication is hydrophilic, has small molecular weight of 250.29 g/mol with moderate volume of distribution (Vd) (0.5–0.7 L/kg). It has low protein binding affinity (< 15%) and is excreted approximately 90% via the kidneys with 40% as unchanged lacosamide [2, 6–8].

Continuous renal replacement therapy (CRRT) is one of the renal replacement therapies and has been chosen for the treatment of acute kidney injury in hemodynamically unstable critically ill patients [9]. Nearly, 50% of critically ill patients with sepsis develop acute kidney injury and subsequently require CRRT for fluid and waste product removal [9]. Most common modalities of CRRT used in ICU settings include continuous venovenous hemofiltration (CVVH) and continuous venovenous hemodialysis (CVVHD). CRRT prescriptions such as modalities, hemofilter types, and effluent flow rates considerably contribute to drug dosing especially drugs with small molecular weight, low Vd and hydrophilic property [9]. Based on the pharmacokinetic characteristics, lacosamide is more likely to be removed via CRRT. Given the variety of CRRT settings in clinical practice, recommended lacosamide dosing in these patients is lacking. In CRRT, applying a higher effluent rate may confer a higher drug clearance [9]. Therefore, finding dosing regimen of the antiepileptics is important as clinicians needs to draw a fine balance between attaining the efficacy and avoiding the side effects.

Changing in pharmacokinetics among critically ill patients include increased volume of distribution, decreased plasma protein binding affinity, and increased drug clearance by CRRT. As mentioned above, drug concentration would be lower and may cause treatment failure [10, 11]. Unfortunately, literature-based lacosamide dosing regimens were mostly gathered from adult case reports [12, 13]. Kalaria and colleagues [14] reported PK parameters of lacosamide in 7 adult critically ill patients receiving CRRT and proposed drug dosing adjustment ranging from 100 to 600 mg/day depending on the various effluent rates of 1–3.5 L/h. When the CRRT with high effluent rates (> 3.5–5 L/h) were prescribed, the lacosamide dose of 600–800 mg/day were recommended.

Furthermore, several lacosamide doses were suggested based on only pharmacokinetic calculation using equations for which most of them were not included the pharmacodynamic (PD) parameters. Recommendations from experts suggested using therapeutic drug monitoring to dose adjust the medication by maintaining trough concentrations within a range of 5–10 mg/L [7, 15] or the area under the concentration–time profile curve (AUC) calculated from PK parameters to be at least 94 mg × h/L [14]. Currently, there is no suggested lacosamide dosing regimens incorporating both PK and PD evaluation among critically ill patients undergoing CRRT.

Our study was aimed to predict the probability of target attainment (PTA) of lacosamide dosing regimens in virtual adult critically ill patients receiving CRRT by applying Monte Carlo simulation (MCS) techniques. We also aimed to identify the correlation between PK parameters and target achievement of lacosamide dosing regimens.

## Methods

Our study protocol was exempted for ethics review in compliance with the Office for Human Research Protections (OHRP Exempt Categories) 45 CFR part 46.101(b) by The Research Ethics Review Committee for Research Involving Human Research Participants,

Group I, Chulalongkorn University (COA No. 072/66).

### Mathematic pharmacokinetic model development

Given the previous published PK studies of lacosamide in adult with epilepsy, they demonstrated that lacosamide characteristic was best-fitted with a one-compartment linear model [6, 14]. The one-compartment mathematical pharmacokinetic models with first-order elimination of critically ill patients with acute kidney disease receiving CRRT were then created to predict lacosamide concentration–time profiles in first 72 h of seizure management [16–18].

Published lacosamide pharmacokinetic parameters in adult critically ill patients with CRRT such as body weight, volume of distribution, non-renal clearance, effluent rates [12–14], and related variability from these patients were gathered to create models of virtual patients with two CRRT modalities. We included the correlations (*r*^2^) between pharmacokinetic parameters as patient’s body weight, non-renal clearance, and volume of distribution into the models to reflect virtual critically ill patients with CRRT in real clinical scenarios. The body weight > 40 kgs was set as a lower limit assuming that all virtual patients are adult.

We included both modalities of continuous venovenous hemofiltration and continuous venovenous hemodialysis in our models. Transmembrane drug clearance [19] was calculated by multiplying effluent flow rate (or dialysate flow rate if the modality is CVVHD) with extraction coefficient. For CVVH, the extraction coefficient is sieving coefficient [12–14].

Since there was no lacosamide pharmacokinetic study in CVVHD to identify reported saturation coefficient (SA), we decided to apply sieving coefficient (SC) values into the CVVHD models. Blood flow rate (Qblood) for all settings was prescribed as 200 mL/min. The equations used in the models were defined as follows [19]:

$${\text{CLHD}}\;({\text{L/h}}) = {\text{SA}}*{\text{Qd}}$$

$${\text{CLHF}}({\text{pre}})\;({\text{L}}/{\text{h}}) = {\text{SC}}*{\text{Quf}}*[{\text{Qplasma}}/({\text{Qplasma}} + {\text{Qreplacement}})$$

$$k = ({\text{CLNR}} + {\text{CLHD}})/{\text{Vd}}$$

$$k = ({\text{CLNR}} + {\text{CLHF}})/{\text{Vd}}$$

where CLHF is transmembrane clearance in hemofiltration; Qplasma is plasma flow rate (Qplasma = Qblood*(1-hematocrit)); hematocrit is 30%; Qreplacement is replacement fluid flow rate (Qreplacement = Quf); CLHD is transmembrane clearance in hemodialysis; Qd is dialysate flow rate; CLNR is non-renal clearance.

According to kidney disease: Improving Global Outcomes (KDIGO) recommended of effluent rates for CRRT prescription, we utilized the effluent rates of 20–25 mL/kg/h in our models [20]. In addition, Srour and colleagues showed that the larger vancomycin dosing regimens were needed in patients receiving high intensity CRRT as of > 3 L/h or > 30 mL/kg/h [21]. Some critically ill patients would also benefit from higher intensity CRRT dosage in terms of solute and volume control goals [22]. Consequently, the effluent rates of 20, 25, 35 mL/kg/h were applied in the models.

Lacosamide dosing regimens in available clinical literature for adult patients with normal renal function and renal impairment [2] were tested in the models. These recommendations were in the range of 100–600 mg/day based on glomerular filtration rates.

### Monte Carlo simulation and probability of target attainment

Following a previously published method [16–18], we applied Monte Carlo simulation technique (Crystal Ball Classroom edition, Oracle) to generate lacosamide deposition of a group of 10,000 virtual patients for each dose to evaluate the probability of target attainment. Pharmacodynamic target of the minimum concentrations of 5–10 mg/L [7, 15]and both area under the concentration–time profile curve targets gathered from published PK studies in a range of 80.25–143 and 143–231 mg × h/L [23, 24] was used to predict the PTA of each lacosamide dosing regimen. The optimal doses were defined as occurring the highest number of virtual patients achieved the pharmacodynamic target with the lowest daily dose to maximize lacosamide efficacy and minimize toxicity. As mentioned earlier, the therapeutic goals for lacosamide in our study were the concentration of 5–10 mg/L or the AUCs of 80.25–143 and 143–231 mg*h/L. Therefore, the PTA is calculated and counted only the concentrations that are within the given therapeutic ranges. For a lower effluent rate of CRRT, the clearance is lower, this provided the higher concentrations of lacosamide that could be above the upper therapeutic goal ranges, resulting in lower % of PTA. Similarly, the higher effluent rate of CRRT, the clearance is higher, the resulted in the lower concentrations of lacosamide, for this reason, the concentrations fell within the therapeutic ranges, which explains the higher % of PTA.

### Statistical analysis

All statistical analyses were performed using SPSS statistical software, version 20 (IBM Corporation, Armonk, NY, USA). We performed log-binomial regression to estimate risk ratios between the proportions of achieving the pharmacodynamic targets and body weight.

## Results

PK parameters in the models gathered from previously published lacosamide studies of adult critically ill patients receiving CRRT are presented in Table 1. In addition, range limits and patients’ body weight utilized in the simulation models are shown in Table 1.

**Table 1:** Demographic and pharmacokinetics simulation parameters of lacosamide in critically ill patients undergoing CRRT [12–14]

Pharmacokinetic parameters	Ranges [limits]
Weight (kg)	75.40 ± 18.40 (40–∞)
*V*_d_ (L/kg)	0.61 ± 0.12 [0.40–1.00]
CL_NR_ (mL/min)	16.60 ± 6.09 mL/min [3.33–26.67]
SC/SA	0.78 ± 0.08 [0–1]

*V*_*d*_ volume of distribution, *CL*_*NR*_ non-renal clearance, *SC* sieving coefficient, *SA* saturation coefficient

Based on the standard effluent flow rates recommended by KDIGO of 20–25 mL/kg/h, lacosamide dosing regimens and PTAs of lacosamide dosing regimens from available clinical resources for both CVVHD and CVVH modalities with three PD targets (trough concentrations and lower and higher AUCs) in critically ill patients receiving CRRT are presented in Tables 2 and 3.

**Table 2:** Average daily PTAs over 72 h in each 10,000 virtual patients receiving 20, 25 and 35 mL/kg/h continuous renal replacement therapy with selected lacosamide regimens using target trough concentrations of 5–10 mg/L and AUCs of 80.25–143 mg*h/L

Selected dosing regimens		Average PTA (%) (trough concentration of 5–10 mg/L)						Average PTA (%) (AUC of 80.25–143 mg*h/L)					
		CVVHD			CVVH			CVVHD			CVVH		
		Effluent rates (mL/kg/h)											
		20	25	35	20	25	35	20	25	35	20	25	35
100 mg q 8 h	In TR	44.22	22.35	3.09	59.64	42.60	14.15	81.04	79.08	60.78	79.84	84.50	81.45
	Above TR	0.03	0.00	0.00	0.07	0.03	0.00	11.51	5.46	0.80	16.30	8.97	2.65
	Below TR	55.75	77.65	96.91	40.29	57.37	85.85	7.45	15.46	38.42	3.86	6.53	15.90
150 mg q 8 h	In TR	88.13	82.52	45.86	85.63	91.85	82.09	26.24	40.16	66.43	16.93	24.71	44.99
	Above TR	7.63	2.39	0.08	13.87	5.68	0.48	73.71	59.56	31.99	83.07	75.26	54.95
	Below TR	4.24	15.09	54.06	0.50	2.47	17.43	0.05	0.28	1.58	0.00	0.03	0.06
200 mg q 12 h	In TR	72.90	48.05	11.75	85.00	74.10	39.44	39.85	56.79	77.92	30.08	42.03	63.17
	Above TR	1.03	0.12	0.00	2.38	0.58	0.00	59.96	42.48	18.35	69.88	57.90	36.36
	Below TR	26.07	51.83	88.25	12.62	25.32	60.56	0.19	0.73	3.73	0.04	0.07	0.47
200 mg q 8 h	In TR	56.35	77.59	84.56	40.03	57.33	85.98	2.48	6.06	20.35	1.04	1.62	5.47
	Above TR	43.62	21.82	3.16	59.97	42.67	13.73	97.52	93.94	79.65	98.96	98.38	94.53
	Below TR	0.03	0.59	12.28	0.00	0.00	0.29	0.00	0.00	0.00	0.00	0.00	0.00
300 mg q 12 h	In TR	71.52	85.88	67.27	56.16	75.02	91.00	1.63	4.56	16.63	0.55	1.26	3.71
	Above TR	28.10	10.40	0.67	43.84	24.94	5.50	98.37	95.44	83.34	99.45	98.74	96.29
	Below TR	0.38	3.72	32.06	0.00	0.04	3.50	0.00	0.00	0.03	0.00	0.00	0.00
250 mg q 8 h	In TR	19.81	42.55	80.58	8.17	18.62	50.15	0.20	0.44	3.78	0.06	0.07	0.26
	Above TR	80.19	57.44	18.06	91.83	81.38	49.85	99.80	99.56	96.22	99.94	99.93	99.74
	Below TR	0.00	0.01	1.36	0.00	0.00	0.00	0.00	0.00	0.00	0.00	0.00	0.00

*PTA* probability of target attainment, *CVVHD* continuous venovenous hemodialysis, *CVVH* continuous venovenous hemofiltration, *LD* loading dose, *TR* therapeutic range. In TR: trough concentration or AUC in the range of 5–10 mg/L and 80.25–143 mg*h/L, respectively, Above TR: trough concentration or AUC greater than 10 mg/L and 143 mg*h/L, respectively, Below TR: trough concentration or AUC less than 5 mg/L and 80.25 mg*h/L, respectively

**Table 3:** Average daily PTAs over 72 h in each 10,000 virtual patients receiving 20, 25 and 35 mL/kg/h continuous renal replacement therapy with selected lacosamide regimens using high target AUCs of 143–231 mg*h/L

Selected dosing regimens		Average PTA (%) (AUC of 143–231 mg*h/L)					
		CVVHD			CVVH		
		Effluent rates (mL/kg/h)					
		20	25	35	20	25	35
250 mg q 12 h	In TR	71.90	71.93	52.32	70.14	75.49	74.00
	Above TR	18.06	9.37	1.60	24.64	15.31	5.13
	Below TR	10.04	18.70	46.08	5.22	9.20	20.87
300 mg LD, followed by 250 mg q 12 h	In TR	70.53	73.44	57.14	64.84	72.94	77.09
	Above TR	22.65	11.72	3.00	31.80	20.58	7.39
	Below TR	6.82	14.84	39.86	3.36	6.48	15.52
350 mg LD, followed by 250 mg q 12 h	In TR	67.16	72.61	63.33	60.99	69.98	78.93
	Above TR	28.17	16.44	3.88	37.04	25.78	10.80
	Below TR	4.67	10.95	32.79	1.97	4.24	10.81
300 mg q 12 h	In TR	52.28	65.50	72.54	42.57	53.62	72.61
	Above TR	46.09	29.94	10.80	56.88	45.12	23.68
	Below TR	1.63	4.56	16.66	0.55	1.26	3.71
150 mg q 8 h	In TR	67.47	56.92	31.77	73.26	70.15	53.87
	Above TR	6.24	2.64	0.22	9.81	5.11	1.08
	Below TR	26.29	40.44	68.01	16.93	24.74	45.05
200 mg LD, followed 150 mg q 8 h	In TR	70.73	62.94	38.16	74.21	73.40	61.67
	Above TR	9.24	4.11	0.54	13.63	7.17	1.74
	Below TR	20.03	32.59	61.30	12.16	19.43	36.59
250 mg LD, followed by 150 mg q 8 h	In TR	73.19	68.83	44.87	73.40	76.55	68.46
	Above TR	12.71	5.94	0.86	18.61	10.58	3.15
	Below TR	14.10	25.23	54.27	7.99	12.87	28.39
200 mg q 8 h	In TR	57.77	67.63	70.65	48.40	58.49	73.77
	Above TR	39.75	26.31	9.00	50.56	39.89	20.76
	Below TR	2.48	6.06	20.35	1.04	1.62	5.47
250 mg LD, followed by 200 mg q 8 h	In TR	51.86	64.79	72.84	42.07	53.85	70.94
	Above TR	46.73	30.92	11.03	57.35	45.03	25.26
	Below TR	1.41	4.29	16.13	0.58	1.12	3.80

*PTA* probability of target attainment, *CVVHD* continuous venovenous hemodialysis, *CVVH* continuous venovenous hemofiltration, *LD* loading dose, *TR* therapeutic range, In TR: AUC in the range of 143–231 mg*h/L, respectively, above TR: AUC greater than 231 mg*h/L, respectively, below TR: AUC less than 143 mg*h/L, respectively

From the three PD targets used in this study, the optimal lacosamide dosing regimens for critically ill patients undergoing CRRT with two commonly used modalities and different effluent rates of 20–25 mL/kg/h and higher effluent rate of 35 mL/kg/h for high volume CRRT were summarized in Table 4.

**Table 4:** Optimal lacosamide dosing regimens for critically ill patients receiving continuous renal replacement therapy with different effluent flow rates and modalities

Effluent flow rates/modalities	Standard PD targets (*C*_trough_ 5–10 mg/L or AUC 80.25–143 mg*h/L)		High PD targets (AUC 143–231 mg*h/L)	
	CVVHD	CVVH	CVVHD	CVVH
20 mL/kg/h	100–150 mg every 8 h		250 mg every 12 h *OR* 250 mg LD followed by 150 mg every 8 h	
25 mL/kg/h	150 mg every 8 h		300 mg LD followed by 250 mg every 12 h	250 mg every 12 h *OR* 250 mg LD followed by 150 mg every 8 h
35 mL/kg/h	200 mg every 8 h	300 mg every 12 h	300 mg every 12 h *OR* 250 mg LD followed by 200 mg every 8 h	350 mg LD followed by 250 mg every 12 h

*PD* pharmacodynamic, *AUC* area under the curve, *CVVHD* continuous venovenous hemodialysis, *CVVH* continuous venovenous hemofiltration

Regarding the effect of body weights on the attainment of PTA targets, it showed that the percentages of attaining the PK/PD targets unidirectionally increased when the body weight is higher in a range of 60–100 kg with *p* value < 0.05 (Table 5). However, the body weight above 100 kg gradually reduced the probability to attain the PK/PD targets in critically ill patients undergoing CRRT compared with those who weigh less than 100 kg. Interestingly, the PTA considerably declined to 70% in the body weight more than 140 kg.

**Table 5:** Effects of body weights on the target attainment of critically ill patients receiving CRRT

Body weights (kgs)	% Patients not attained the targets (*N*)	% Patients attained the targets (*N*)	Unadjusted RR (95% CI)	*p* value	Adjusted RR (95% CI)	*p* value
< 60	42 (828)	58 (1142)	0.180 (0.165–0.198)	< .001	0.714 (0.412–1.236)	.229
60–70	17 (384)	83 (1924)	0.823 (0.739–0.916)	< .001	1.803 (1.036–3.138)	.037
71–80	7 (147)	93 (2088)	2.526 (2.144–2.976)	< .001	4.56 (2.583–8.053)	< .001
81–90	2 (33)	98 (1545)	7.972 (5.668–11.210)	< .001	14.345 (7.546–27.273)	< .001
91–100	0 (3)	100 (968)	51.405 (16.592–159.266)	< .001	97.100 (27.678–340.650)	< .001
100–110	1.5 (8)	98 (511)	9.778 (4.908–19.480)	< .001	19.463 (8.086–46.846)	< .001
110–120	3.4 (8)	97 (230)	4.355 (2.200–8.621)	< .001	8.925 (3.726–21.376)	< .001
120–130	12 (13)	88 (92)	1.162 (0.697–1.938)	.564	2.423 (1.148–5.113)	< .001
130–140	9 (4)	91 (42)	1.656 (0.648–4.228)	.292	3.450 (1.167–10.203)	.020
> 140	30 (9)	70 (21)	0.477 (0.276–0.826)	.008	1	.025

## Discussion

This is the first simulation study applying MCS technique to evaluate dosing of lacosamide for seizure management in critically ill patients. We gathered all necessary PK parameters from previous published PK studies conducted in adult critically ill patients receiving CRRT, including body weight, Vd, non-renal clearance, and SC/SA to evaluate and establish dosing regimens [12–14]. We modeled our simulation using KDIGO recommended effluent rates of 20–25 mL/kg/h, and also applied the higher intensive effluent flow rate of 35 mL/kg/h into the model [21, 22]. All necessary parameters were incorporated into pharmacokinetic models to predict lacosamide disposition in critically ill patients receiving CRRT for 72 h. The correlations between pharmacokinetic parameters were applied in the models to create population-specific virtual patients.

As mentioned, critically illness have an impact on drug dosing in these patients. Volume of distribution of hydrophilic agents tends to be increased due to fluid accumulation and hypoalbuminemia [9, 10]. Larger Vd causes subtherapeutic drug concentrations and may lead to treatment failure. Giving higher doses can be suggested to achieve PK/PD targets. However, the Vd values gathered from the previous studies in our model was 0.61 ± 0.12 L/kg [12–14] which was similar to the healthy volunteers of 0.6 L/kg [2, 25]. Therefore, lacosamide dosing regimens may not be greatly affected in critically ill patients due to the comparable Vd between critically ill and normal population.

We assumed that the critically ill patients with CRRT have a renal clearance of 0 mL/min in our model. Therefore, total lacosamide clearance was derived from two major factors, including CRRT and non-renal clearance. CRRT clearances (CVVH and CVVHD clearance) were calculated via the equations mentioned in Method section. Non-renal clearance retrieved from previously published pharmacokinetic studies was 16.31 ± 6.09 mL/min [12–14] and was then utilized in the models. As for the non-renal clearance in healthy subjects, it was approximate to be about 60% of total body clearance of 2 L/h [8, 25], which is 20 mL/min, and is similar to the values extracted from the available pharmacokinetic studies in critically ill patients receiving CRRT. Therefore, the contribution of non-renal clearance in lacosamide dosing adjustment in critically ill patients is relatively minimal.

The typical lacosamide dosing regimens were recommended for 200–400 mg daily. The dose as high as 600 mg/day have been utilized and may be beneficial in some patients [2, 8, 25, 26]. Unfortunately, no standard guidelines of therapeutic drug monitoring of lacosamide exist due to the lack of the exact correlation between serum concentrations and therapeutic efficacy. May and colleages [27] provided the correlation between serum and cerebral spinal fluid (CSF) samples from 21 patients receiving lacosamide for epilepsy management. The optimal correlation was described as the mean CSF-to-serum ratio of 0.897 ± 0.193 in the daily dose range of 50–600 mg. They suggested the serum concentration of lacosamide may be an important indicator of central nervous system concentration to estimate therapeutic efficacy [27]. Therapeutic drug monitoring of lacosamide was proposed by some experts with the lacosamide concentration ranging from 5 to 10 mg/L [7, 15]. Laveille and colleagues proposed that the trough concentration producing half the maximum seizure frequency reduction (EC_50_) was 4.6 mg/L [28]. Therefore, we infer that the minimum concentration of approximately 5 mg/L is required to attain seizure control. Moreover, Kropeit and colleagues [23] conducted a pharmacokinetic study to reveal lacosamide concentrations after receiving oral and IV lacosamide of 200 mg single dose. The mean maximum concentration was 5.95 + 1.49 mg/L with AUC of 80.25 mg*h/L and there were not statistically different between both oral and IV administrations. Horstmann et al. [24] also conducted a pharmacokinetic study using 400 and 600 mg of lacosamide. The mean concentrations were 8.7 + 1.8 and 14.3 + 2.3 mg/L, respectively. The AUCs of the 2 doses were reported as 143 + 27 and 231 + 49 mg*h/L, respectively [24]. Based on the pharmacokinetic studies and recommendations from the experts, we decided to adopt the concentration range of 5–10 mg/L as our targeted trough concentrations and use into our models to represent the lacosamide doses of 200–400 mg daily which are doses recommended by clinical literature for general patients. As presented in previous pharmacokinetic studies, the AUC target range of 80.25–143 reflects the dosing regimens of 200–400 mg daily. In addition, some patients who need additional benefits from increasing the dose up to 600 mg/day, the higher AUC range of 143–231, which equates to the doses of 400–600 mg/day, may be required. Therefore, we decided using both standard and higher AUCs of 80.25–143 and 143–231 mg × hour/L to ensure the accuracy of our models in addition to the trough concentrations as pharmacodynamic targets. Our results showed that regardless of the standard AUC target or trough concentration ranges used, the optimal dosing recommended were similar, as shown in Table 4. While using the higher AUC target range, larger dose is required to achieve the pharmacodynamic target, as shown in Table 4.

The maximum dose of lacosamide of 400 mg and 600 mg are recommended in United States and European Union, respectively. Some suggested doses in Table 4 especially in high pharmacodynamic target group exceed the maximum dose of 600 mg/day. Ben-Menachem and colleagues [29] explored the long-term safety and tolerability of lacosamide monotherapy in patients with epilepsy in the dose range of 200–600 mg/day. The daily lacosamide dose up to 600 mg was defined to be generally well-tolerated [29]. Therefore, when the lacosamide dose above 600 mg is needed to control seizure in patients who require high pharmacodynamic target and receiving CRRT, closely monitoring of lacosamide adverse events is absolutely recommended in patients with epilepsy. In addition, there was a pharmacokinetic study conducted by Cawello and colleagues [30] to identify the bioequivalence of intravenous and oral lacosamide formulations. 200 mg of oral and IV infusion lacosamide formulations were given to healthy volunteers. It showed that bioequivalence was demonstrated for IV and oral formulations in terms of AUC and maximum concentration (Cmax) [30]. Direct conversion from oral to IV lacosamide, or vice versa, is possible. However, pharmacokinetic changes in critically ill patients are challenging for drug dosing especially in absorption process [31]. Changes in gastric pH, delayed gastric emptying, drug–food interactions, and/or altered efflux transporter activity play major contributions to unreliable absorption. Therefore, the IV route of administration is strongly recommended [31].

Presently, the recommended dosing regimens of lacosamide for patients receiving CRRT are only based on two case reports [12, 13]. Franquiz and colleagues [13] presented a case study of the patient with status epilepticus and multiorgan failure undergoing CRRT with the effluent rate of 2.3 L/h who was prescribed 400 mg/day of lacosamide. Lacosamide was effectively removed via CRRT with the sieving coefficient was 0.8 ± 0.06. Vd and non-renal clearance were identified as 0.7 L/kg and 13.42 mL/min [13]. The second case report was published by Wieruszewski and colleagues [12]. The patient developed nonconvulsive status epilepticus and received CRRT with the same effluent rate and was prescribed lacosamide 400 mg intravenously daily. The Vd, non-renal clearance and sieving coefficient were reported as 0.69 L/kg, 25.20 mL/min and 0.69 for which they were similar compared to the first case report [12, 13]. Notably, their dosing recommendations were done based on only PK parameters without utilizing pharmacodynamics outcomes, while in our study, we applied both PK and PD, and combined the MCS technique to amplify the outcomes of efficacy and toxicity. Consequently, our recommended maintenance doses of 100–150 mg every 8 h with the standard KDIGO-recommended flow rates were different to the regimen from both case reports of 200 mg twice daily.

Recently, Kalaria and colleagues [14] conducted a pharmacokinetic study of lacosamide use in critically ill patient receiving CRRT to establish a lacosamide dosing protocols from PK parameters in 7 critically ill patients undergoing CVVH. The average of SC, Vd and non-renal clearance were 0.79, 0.58 L/kg and 15.50 mL/min, respectively [14]. They proposed the protocol of lacosamide dosing regimens for patients receiving CRRT depending on effluent flow rates and lower or higher exposure dosing regimen [14]. The dosing protocol was based on PK parameters and a PD target of 94 mg × hour/L. In our study, we included both PD targets (trough concentration, standard and aggressive AUCs) and applied MCS to define the optimal dosing regimens [7, 15, 23, 24]. Our results showed when the effluent rate is higher as 35 mL/kg/h, a higher lacosamide dose of 200 mg three times daily was required. Similarly, if the higher AUC is chosen to better seizure control, the larger doses is needed. Consequently, we recommended the optimal dosing regimens for adult critically ill patients CRRT based on effluent rates and PD targets in Table 4.

In addition, we tested the effect of body weights on achieving the PTAs using log-binomial regression to define risk ratios, the body weight range of 60–100 kg was found to have a good correlation to attain the PTA target compared to patients with more than 100 kg. The finding was aligned with the results from two landmark randomized controlled studies [4, 5] of lacosamide to evaluate the efficacy and safety for partial-onset seizures. Both trials showed the similar results in significant improvement of seizure control when lacosamide was prescribed as 400–600 mg daily in the patients with average body weights in the range of 74.5–81.0 kg [4, 5]. Consequently, when lacosamide was used in these patients, those three factors as body weight, desired pharmacodynamic target and effluent flow rate should be considered for clinicians to determine drug dosing modification in patients receiving CRRT, especially in patients weighed > 100 kg.

Limitations of our study were listed as follows: (1) we defined the optimal dosing regimens using available published PK studies, such as body weights, non-renal clearance, sieving coefficient and volume of distribution. All combined pharmacokinetic data were only from adult patients. Therefore, our recommendation should be applied for the patients who match our assumptions. (2) our dosing recommendations are suggested in anuric patients. If lacosamide is used in patients with higher renal clearance, the dose should be adjusted. (3) the pharmacokinetic changes in critically ill patients are dynamic and depends on individual patient conditions, we recommend closely monitoring of lacosamide concentrations. (4) To our knowledge, there is no standard guideline for lacosamide therapeutic drug monitoring and desired target lacosamide concentrations. However, the trough concentration producing half the maximum seizure frequency reduction was 4.6 mg/L as presented by Laveille and colleagues [28]. In addition, Svendsen and colleagues [32] conducted a pharmacokinetic study using therapeutic drug monitoring data of lacosamide from The Norwegian Prescription Database. They revealed that the average lacosamide concentration in patients with modest and good efficacy should be at least 5.71 mg/L, while non-responders had an average lacosamide concentration as 4.65 mg/L [32]. This concentration is aligned with our lacosamide target range of 5–10 mg/L. Hence, monitoring of clinical conditions would be required to assure lacosamide efficacy and toxicity. Clinical validation of this finding is needed.

In conclusion, we suggested optimal lacosamide dosing in critically ill patients undergoing CRRT depending on different modalities and the pharmacodynamic targets in Table 4. The effluent rate as 35 mL/kg/h required higher lacosamide doses. Three main factors as total clearance, volume of distribution, and body weight are responsible for lacosamide dosing modification to achieve the pharmacokinetic and pharmacodynamic targets. Pharmacokinetic changes in critically ill patients owing to pathophysiologic variability need to be aware for lacosamide prescription to avoid treatment failure or drug toxicity. Moreover, larger doses than our recommendations with closely drug monitoring would be considered in the patients with body weight more than 100 kg.

## Data Availability

The data sets used and/or analysed during the current study are available from the corresponding author on reasonable request.

## Abbreviations

AUC: Area under the concentration–time profile curve
CLHD: Transmembrane clearance in hemodialysis
CLHF: Transmembrane clearance in hemofiltration
CLNR: Non-renal clearance
Cmax: Maximum concentration
CSF: Cerebral spinal fluid
CVVH: Continuous venovenous hemofiltration
CVVHD: Continuous venovenous hemodialysis
CRRT: Continuous renal replacement therapy
EC_50_: Half the maximum seizure frequency reduction
h: Hour
ICU: Intensive care unit
KDIGO: Kidney Disease: Improving Global Outcomes
kg: Kilogram
L: Liter
MCS: Monte Carlo simulation
mg: Milligram
mL: Milliliter
OHRP: The Office for Human Research Protections
PD: Pharmacodynamics
PK: Pharmacokinetics
PTA: Probability of target attainment
Qblood: Blood flow rate
Qd: Dialysate flow rate
Qplasma: Plasma flow rate
Qreplacement: Replacement fluid flow rate
Quf: Ultrafiltration flow rate
SA: Saturation coefficient
SC: Sieving coefficient
Vd: Volume of distribution
